# Reviews of Books

**Published:** 1924-02

**Authors:** 


### REVIEWS OF BOOKS.

### THE ALIENIST AND THE CRIMINAL.

**Insanity and the Criminal Law.** By WILLIAM A. WHITE, M.D. (New York, The Macmillan Company. 12s. net.)

Dr. White is the superintendent of St. Elizabeth's Hospital for the Insane at Washington, and he writes with a long experience of the subject with which he deals. The problem of " criminal responsibility " is always with us, but recent events have brought it once again into special prominence. Although the problem is essentially the same in England as in America, the manner in which it is practically determined differs in the two countries. In some ways our methods appear preferable. For example, Dr. White complains with some bitterness, and with apparent justice, of the way in which expert evidence offered by alienists in criminal trials is treated in his country. With us there is a body of experts, the officers of our prison medical service, whose evidence is regarded as quite impartial, is treated with much respect, and is usually accepted without question.

It is impossible to consider this question without a review of the nature of crime, and of the basis and functions of the criminal law. Dr. White deals with these matters briefly but adequately. He points out that " criminal conduct " is an abstract term applied to socially destructive tendencies manifested by individuals, and that criminal law is intended to combat these tendencies, and he holds that the basis of punishment can, ultimately, be traced to the primitive desire for vengeance. However much we may rationalise, we can never wholly escape from this retaliatory instinct. Dr. White gives us a number of illustrative cases, in which he considers that the accepted legal criteria of responsibility worked unsuccessfully in practice. Most psychiatrists would agree with his view on the evidence put before us in this book; but the real difficulty occurs when we make an attempt to frame better criteria. The present tests are concerned with knowledge of the " nature and quality " of an act, and knowledge of the difference between right and wrong. That these tests are unsatisfactory is admitted by lawyers as well as by the medical profession. Dr. White proposes, as a substitute, that the test should be whether at the time of the act the man was " suffering from mental disease, and by reason of such mental disease did not have the particular state of mind that must accompany such act in order to constitute the crime." We doubt whether this is any real improvement. It appears likely that just as many disputes would arise under this new test as we have now with our present tests.

Dr. White urges that the sole function of the jury should be to decide whether or not the man had committed the act with which he is charged. What should be done with the man would be decided afterwards, and in this decision the psychologist would have the final word. There is much to be said for this plan, but it will be a long time before our legal authorities agree to it. Even now lawyers are very jealous of the incursions which psychology has been making into the legal preserve. Dr. White also suggests that schools of criminology should be established in which judges and all who deal with offenders should be obliged to take a course. There is much ignorance of the psychological principles which are involved in criminology, and such schools, even if attendance at them were not compulsory, would do something to dispel this ignorance. A tentative attempt at such a school has recently been made by Birmingham University, but attendance is at present confined to medical practitioners.

The whole book is most excellently written, and we can cordially commend its perusal to all who are interested in these fascinating and vital problems.

### THE CONTROL OF DRINK.

**Drink in 1914-1922: A Lesson in Control.** By ARTHUR SHADWELL, M.A., M.D., LL.D., F.R.C.P. (Longmans Green and Co. 1923.)

Dr. Shadwell can always be trusted to talk sense on the subject of drink, which has such a bad influence in driving most of those who discuss it to extremes. The late war, or rather, the measures of control which were taken while, and for a short time after, it lasted, have provided him with just such a theme as he is best qualified to expound. Apart from the immediate occasion there are all the materials here for an instructive treatise on manners and morals. At one time a writer who thought as Dr. Shadwell does, would probably have given the world what he had to say in the form of a comment on the last half dozen verses of the thirty-first chapter of Ecclesiasticus. His final conclusions are substantially in agreement with the wisdom of Jesus, the Son of Sirach, who laid it down that wine is good, but that excess is an evil. The problem to be solved is how to abate the excess, without provoking reaction and revolt by too drastic an interference with established customs, and a tyrannical degree of compulsion. This has been the purpose of all licensing laws and was the very pressing task imposed on the British, and, indeed, other Governments, during the war.

It will stand to our credit that the measures taken not only had the effect desired at the time, but also did not a little which promises to be permanently beneficial. One must know how to distinguish and divide, how to allow for all influences. Therefore, it would be grossly uncritical to say that the entire prohibition enforced in Russia was alone responsible for the hideous anarchy which has overwhelmed that country. But it is a manifest fact that the, no doubt, well-meant, and—seeing that so large a part of the Tzar's revenue was drawn from the sale of drink—the unselfish, prohibition imposed on the trade, has not had the least effect in improving the morals or the sense of the people. The moderate policy of the British Government seems almost sure to promote a lasting increase in sobriety. How it was all begun, how it was developed, what particular restrictions were applied, and what palliatives were used, is set forth by Dr. Shadwell with an admirable lucidity of statement and a wealth of evidence which cannot be properly appreciated except by a careful reading of the book. Nine chapters are devoted to a statement of the case, to an account of the machinery created for the execution of the policy, to various aspects of the whole, such as "The Provision of Meals," "State Ownership," "Output, Taxation, and Dilution," and their effects. A chapter of "Conclusions" draws the moral, and then comes a well-equipped appendix, or body of appendices, made up of documents and statistics. Taken together, the chapters and the appendices supply a contribution to the history of the war of the highest value. And they do more. They show how the good then obtained may be preserved.

It is far from being the least of the merits of this treatise that a definite view is stated, and supported by means of an abundant quotation of details. Dr. Shadwell's doctrine, as he may fairly call it, is that the true method of reform is to be found in the removal of temptations to excess, and in the provision of alternatives in places where drink is sold. It is a favourite contention of his that the last hour of sale at night is the time when drunkards are made. The shortening of the open hours, and the interruption of a continuous day of sale have done much for sobriety, and will continue to have the same wholesome effect. Every resident in a great town must have learnt, if he keeps his eyes about him, and his ears open, that an earlier closing hour, a later opening time, and the break in the afternoon, have notably diminished the amount of tipsy noise in the streets. Police statistics show the same results. Dr. Shadwell will have nothing to say in the way of agreement with those—for there are some—who denounce all restrictions. With his usual independence of cant, he acknowledges that there are many of the people who are not to be trusted to drink in moderation. They are not all vicious. Many of them, even most of them perhaps, are weak. They will not exceed when they are protected against temptation.

At the same time it is clearly proved that when restriction goes beyond a point not easily to be defined, but soon detected in practice, there is rebellion, and pure harm is done. Dr. Shadwell quotes one telling example of what comes of going too far. Drawing on his extensive knowledge, he cites the consequences of exaggerated restriction in Sweden. For a time the reduction in the amount of strong drink which could be lawfully supplied, was accompanied by a reduction in the number of cases of drunkenness reported by the police. When the Government, encouraged by this reduction, approached closely to Prohibition, cases of "police drunkenness" doubled in a year. The police had to report that people had taken to methylated spirits and to illicit distilling in their own houses, and that to such an extent as to be beyond control. English good sense, of which we now and then talk too much, but which does exist, kept our Government from falling into this error. It was as Dr. Shadwell shows, helped not to wander from the safe middle path by timely expressions of public opinion. He has suggestions to make as to changes in the licensing laws, which appear to us to be very sensible. They should be read on his own pages.

### OBSTETRICS.

**Obstetrics: A Text-book for the Use of Students and Practitioners.** By J. Whitridge Williams, Professor of Obstetrics, Johns Hopkins University; Obstetrician-in-Chief to the Johns Hopkins Hospital, Baltimore, etc. (D. Appleton and Co., New York and London. 5th Edition. Price 40s.)

This excellent and popular work on Obstetrics, which in its present edition extends to 1,074 pages, may truly be described as monumental. It has been thoroughly revised and contains 17 full-page coloured plates besides the large number of nearly 700 illustrations. Radical changes, bringing the contents well up-to-date, have been made in the sections dealing with ovum development, the toxœmias of pregnancy, indications for Cæsarian section, and others. Throughout the book the theory and practice of obstetrical science are dealt with side by side, the practical aspects of the subject being so treated as to make the information equally useful to the practitioner and the student. The standard of obstetrical practice follows closely on the lines laid down by the Johns Hopkins Hospital as carried out in its Woman's Clinic, and forms a sound working basis, subject, of course, to modification in individual cases.

In the Preface a note of warning is sounded regarding the danger of regarding labour as a pathological rather than a physiological process, and while the technique of all the various operative procedures is described in detail, the greatest possible conservatism is insisted upon, consistent with the welfare of mother and child. This new edition should prove fully as popular as those which have preceded it.

### THE RISE OF IMPERIAL TRADE.

**The Romance of Colonisation: Being the Story of the Economic Development of the British Empire.** By Arnold Wright. (Melrose. 15s. net.)

Mr. Wright has chosen a picturesque title for his handsomely produced book, and he writes with the chastened picturesqueness of a man dealing with a romantic subject of the very first practical moment. He has long been a diligent student of the history and progress of the Britains beyond the seas, and his book on *Early English Adventurers in the East* will have taught his readers to expect a highly attractive presentation of facts. He has, indeed, given us a sketch, necessarily rapid, but thoughtful and founded on data much of which is unfamiliar, of the growth of imperial trade which could not easily be bettered. From first to last he is readable with pleasure, which is far more than can be said of most books in this category.

Much space is perforce given to that tremendous Trade and Navigation Act with which Cromwell sought to smash Holland's determined attempts to secure predominance in external trade. This Act ordained that no goods might be imported into England, Ireland, or any of the plantations from Asia, Africa or America save in an English ship, or in a ship belonging to the English plantations, and with a crew of at least one-half of which were English. Furthermore, no goods could be imported from any European country save in a ship of that country or an English ship. Dutch exasperation at a policy which would obviously soon be fatal to their mercantile marine led to a war in which Holland was eventually discomfited, and at the Restoration the Act was so strengthened as to forbid exports as well as imports in any but English ships. These laws did good work in their day. They countered the Dutch menace, but they were kept on the Statute Book far too long—two hundred years—and Mr. Wright is probably not far wrong when he suggests that our supremacy in sea-borne trade would have been equally well secured by the national aptitude of the English for seamanship. Moreover, there is little doubt that they laid the seeds of American Independence. The New England colonists systematically and flagrantly evaded them. Nearly a century before 1776 there was rampant disaffection, and there has rarely been a clearer chain of cause and effect than that which connects the Trade and Navigation Acts with the American Revolution:—

> The cause of offence was not so much the endeavour to extort taxes from the colonists as the sense of humiliation inflicted upon them by a mercantile system which dictated not only what markets they should take their goods to, but the character of the industries upon which they should engage in their own land. That there was another side to the question—that the Mother Country enforced upon herself the same restrictive system which she imposed upon others, and that she bore the very heavy charges of defending the colonies from outside aggression, and, therefore, was justly entitled to financial compensation—was neither here nor there in a dispute which touched the pride and independence of the colonists.

The Stamp Act, in short, was the excuse for, rather than the cause of, the Revolution. We have dwelt upon this event, one of the most important in the history of the world, because historians have been too much inclined to ignore the long-standing and deep-seated causes of friction, and because the violent separation between England and America is the most conspicuous instance in history of the enormous influence of trade and mercantile systems upon the destinies of peoples. In Mr. Wright's book, however, this, although the greatest, is only one of many episodes. He is largely concerned with tobacco and sugar, indigo and rubber, with slavery and the system of white bondsmen with which it was sought to develop the West Indies. At a moment when questions of inter-Imperial trade relations seem likely once more to cause serious difficulties his admirably-written and carefully-marshalled volume ought to be in the hands of everyone who appreciates the much disregarded fact that the roots of the present lie in the distant past.

### THE NEW "BURDETT."

**Burdett's Hospitals and Charities, 1924.** The Year-Book of Philanthropy and the Hospital Annual. Founded by the late Sir Henry Burdett, K.C.B., C.V.O. (The Scientific Press, Ltd., 28-29 Southampton Street, Strand, W.C. 2. 17s. 6d. net.)

In the preface to this indispensable book of reference for 1924 we are reminded that with its present issue the work enters upon its twenty-fourth year of publication. Long ago it established its right to a permanent place on the office-table of most hospitals and benevolent institutions, and with each succeeding year its usefulness has been more than maintained. It has indeed increased by leaps and bounds. In its present extended form, containing 100 extra pages of information, it is better than ever. We are reminded in the Preface that hospital finance, both in London and the Provinces, has distinctly improved. In 1920 the aggregate deficit of 113 London hospitals was £383,000, and in 1921 £210,000; in 1922 it fell to £174,000. This is extremely encouraging, and even better results were achieved by the 666 Voluntary Hospitals outside London, which in 1921 showed a total deficit of £419,000, but in 1922 reduced it to £75,000. Much good work has been done for the hospitals by the many contributory schemes which, of late, have made considerable progress, and of these the Hospital Saving Association is a conspicuous example. Full particulars are given of this scheme, which, though started only in 1922, already promises to be one of the best yet devised for the support of hospitals and the provision of necessary beds for the hitherto neglected lower middle-class in sickness.

In this issue, Irish hospitals and institutions now occupy considerably more space than hitherto, and fall under their proper divisions of "Northern Ireland" and the "Irish Free State." Overseas hospitals also have received far more detailed attention, which has naturally called for pruning in other directions. Hospitals of the United States have therefore been eliminated to make room for an all-British list, an alternative regretted by the compilers, but necessary to keep the size of the book within reasonable limits. As far as possible, and as the result of much labour in the classifying and arranging of material, the contents of the volume have been brought well up-to-date, and will prove a most trustworthy source of information to its users throughout 1924.

### IN MEAN STREETS.

**The Old Doctor.** By Frank G. Layton, M.R.C.S. L.R.C.P. (Cornish, Birmingham. 4s. 6d. net.)

"The Old Doctor" practised among the poor of Walsall, the big South Staffordshire town where Sister Dora lived and worked. We need be under no doubt about the locality. Not only does Dr. Layton himself practise there, but in an occasional moment of forgetfulness he mentions the actual names of streets in Walsall. He has written a very human, if a rather sad, book, full of the self-sacrifice of medical men tending the poor in mean streets, with little or no pay, in the days before the coming of Health Insurance. It is a fine and noble company that Dr. Layton has drawn, with their ever readiness to help people as miserably ignorant as they are miserably poor, with all the pathetic belief of their class in a bottle of medicine. But when we come upon the "eminent consultant" of the place who prescribes a sixpenny bottle of stuff for a girl with a gangrenous appendix calling for instant operation, we learn something of the pestilent type of medical man which is, happily, nearly extinct. These same people who pin their faith to medicine have a superstitious horror of even the most necessary operation—"cutting and carving" is to them the abomination of desolation.

The moral of Dr. Layton's book is that the General Practitioner has a hard life if he means to lead an honest one. But he is quick to show us its compensations, and the illimitable good that can be worked by the right kind of man, especially in an industrial community where back streets hide so much of the poverty and squalor that are the parents of disease. He is an intense believer in the panel system—so long as it is not spoiled by red tape. When it began every doctor in "Wonston" "went on the panel, thankful to make an end of the old club system." Yet "Old Luke," as he calls his hero, recognises that no medical service will ever be thoroughly effective so long as slums persist:—

"It'll cost a lot of money," he said, suddenly, one hot evening when he was taking a late stroll with Trelawny. "What will?" "Pulling down all these houses and building new ones." He pointed through an entry which led down into a court. The street in which they stood was grey and grimy, littered with every conceivable sort of refuse; the court was foul, squalid, almost inconceivably dismal. But there were a dozen houses in it, and one standpipe for the supply of water. There were several ashpits, all exposed to the rain, each one a fermenting mass in which flies bred in millions.

There are still thousands of such streets and courts scattered about the country, and the first aim of every social reformer should be to get rid of them. The General Practitioner whose work lies in industrial areas will read Dr. Layton's clever little book with curiosity and pleasure, and it will do no harm if that other G. P., the general public, read it as well.

### MISCELLANEOUS NOTICES.

### The Mother's Task.

The Mothercraft Manual. By Mabel Liddiard. (J. A Churchill. 3s. 6d. net.)—Of the making of this type of book there would appear to be no end, and it is to be presumed, therefore, that the modern mother is avid of such literature. Certainly Miss Liddiard's manual is an excellent specimen of its kind—clearly arranged, well illustrated and full of sensible and robust advice, with particularly valuable chapters on feeding. Dr. Fairbairn contributes an Introduction in which he speaks of the remarkable work done by Miss Liddiard while Matron of the Mothercraft Training Society.

### Extrauterine Pregnancies.

Death of the Extrauterine Fœtus. By James Oliver, M.D.—In this interesting paper, reprinted from the *New York Medical Journal*, the author considers that the early implantation of the human product of conception, wherever it takes place, is effected by chemiotaxis. In former days, when abdominal section was not adopted, the results of extrauterine pregnancies were allowed to disintegrate and undergo absorption. In cases where the full time fœtus dies at a definite and fixed time, as usually occurs, Dr. Oliver believes that this is due to a lack of oxygen and mineral substances owing to the limit of the functioning power of the placenta having been reached, and the cells concerned having lost their selective powers.

### Dental Caries.

Oral Hygiene. By J. Sim Wallace, D.Sc., M.D., L.D.S. (Ballière, Tindall and Cox. 5s. net.).—This book contains four papers, three of which have been previously published, and the whole forms a composite exposition of the author's theories on the cause and prevention of dental caries. Although some of the comments and criticisms made upon the views of others are rather provocative in tone, the arguments put forward by Dr. Wallace in favour of his own theories are by no means unsound. Essentially he would remind us that the function of ptyalin is not necessarily to aid digestion, but more probably to remove carbohydrate débris from the cracks and crevices of the mouth in general, and the teeth in particular. This carbohydrate residue may well be the primary cause of caries, whatever may be the secondary functions the various vitamin deficiencies, the activities of bacteria and leptothrix filaments and other suggested factors ultimately play. In any case this little book should be read by all those interested directly or indirectly in the prevention of dental disease. It may give them many new ideas.

